# A TinyML Deep Learning Approach for Indoor Tracking of Assets †

**DOI:** 10.3390/s23031542

**Published:** 2023-01-31

**Authors:** Diego Avellaneda, Diego Mendez, Giancarlo Fortino

**Affiliations:** 1 School of Engineering, Electronics Engineering Department, Pontificia Universidad Javeriana, Bogotá 110231, Colombia; 2 Department of Informatics, Modeling, Electronics and Systems, University of Calabria, 87036 Rende, Italy

**Keywords:** RSSI, iBeacon, deep learning, TinyML

## Abstract

Positioning systems have gained paramount importance for many different productive sector; however, traditional systems such as Global Positioning System (GPS) have failed to offer accurate and scalable solutions for indoor positioning requirements. Nowadays, alternative solutions such as fingerprinting allow the recognition of the characteristic signature of a location based on RF signal acquisition. In this work, a machine learning (ML) approach has been considered in order to classify the RSSI information acquired by multiple scanning stations from TAG broadcasting messages. TinyML has been considered for this project, as it is a rapidly growing technological paradigm that aims to assist the design and implementation of ML mechanisms in resource-constrained embedded devices. Hence, this paper presents the design, implementation, and deployment of embedded devices capable of communicating and sending information to a central system that determines the location of objects in a defined environment. A neural network (deep learning) is trained and deployed on the edge, allowing the multiple external error factors that affect the accuracy of traditional position estimation algorithms to be considered. Edge Impulse is selected as the main platform for data standardization, pre-processing, model training, evaluation, and deployment. The final deployed system is capable of classifying real data from the installed TAGs, achieving a classification accuracy of 88%, which can be increased to 94% when a post-processing stage is implemented.

## 1. Introduction

The expansion and continuous improvement of smartphones and personal electronic devices have resulted in the remarkable development and growth of many Information and Communications Technology (ICT) industries. Indoor positioning technology has developed to support a variety of services making use of the sensors and protocols embedded in mobile devices. After the emergence of iBeacon [1] and Eddystone [2], attempts to detect position in indoor environments using Bluetooth have increased compared to WiFi-based techniques, mainly because Bluetooth is designed to operate with low power consumption [3,4,5,6].

Indoor positioning has multiple applications, and has been the subject of study in different industries, location-based marketing being one of the areas that has led to the most research on these systems. The goal of location-based marketing is to provide customers with a personalized experience and to provide special offers depending on their location as well as information about products or services. This type of solution, along with payment integration through notifications or tracking of consumer behavior, provides information that can improve the end customer experience [7].

In this work, an IoT-based system was realized and Machine Learning (ML) was used to estimate the position of assets, objects, people, or animals carrying a TAG device in indoor spaces. For such a system, different methodologies, architectures, and frameworks must be considered in order to propose an optimal solution for such an application [8]. As trilateration is not quite suitable for indoor location estimation, this project considers the fingerprinting technique, in which an intelligent system collects information from RSSI (Received Signal Strength Indicator) levels from the wireless communication between the object to be located and different access points, then associates these collections of RSSIs (fingerprints) to a certain location in space [9,10,11]. Through Bluetooth Low Energy (BLE) technology, a TAG attached to the object to be located sends periodic beacon messages, which are scanned in a fixed time window by *Scanning Stations* deployed in the environment under study. The scanning stations consolidate and send the information to a server, where a previously trained Deep Learning (DL) model estimates the position of the object utilizing the fingerprinting approach.

The main objective of the IoT indoor positioning system is to allow the identification and tracking of assets in such a way that it is possible to generate presence or absence alerts. This goal is achieved with the development of different elements of an IoT architecture, involving: the (i) implementation of fully customized electronic devices (hardware and firmware) for the scanning stations and mobile TAGs; (ii) a Bluetooth communication layer between the TAGs and the scanning stations; (iii) the centralization of the information to a local MQTT broker; and (iv) the storage and edge computing layers. The project first included a training stage, during which a location dataset was acquired and labeled for later use as the input for training the neural network, followed by a second deployment and operation stage.

Despite many different efforts for indoor positioning, there is no established standard for these techniques. Important efforts have been made to propose a general simulation-driven methodology for IoT systems to enable data mining and machine learning applications, with particular focus on the edge, for taking effective actions in constrained and dynamic IoT scenarios [12]. The work in [13] presents a patent that is specifically oriented in the field of radio frequency and the deployment of multiple devices in an indoor environment, aiming to create a navigation infrastructure for marketing, consumer analysis, emergency situations, transportation, and parking purposes, among others. The proposed solution is based on the application of ML techniques that can infer the status and positioning of the user depending on the input flow of information from the TAGs, which are electronic devices that are installed in the local area, either indoors, outdoors, or at points of interest, and that emit a unique identifier via Bluetooth. The work presented in [14] proposes the K-Nearest Neighbor (KNN) algorithm to study the accuracy of fingerprinting positioning and compares its performance with other approaches such as Support Vector Machines (SVM), Random Forest (RF), and Multi-layer Perceptron (MLP).

The authors in [15] designed an ML method to reduce the standard deviation of the positioning estimation error and increase the probability that the estimated distance is within 2.5 m of the actual distance. The application of RSSI methods addresses the difficulties in localization in environments with obstacles. While significant improvement in accuracy can be achieved by ML techniques, the computational cost is controllable with customization in environmental and device setup [16]. In a three-dimensional space, BLE-based spaces can provide accurate location as long as the boundaries of each of the devices are predefined to infer the location from the highest signal strength in the coordinates. Lin et al. proposed the LNM scheme, an approach that utilizes the neighbor relative received signal strength to build a fingerprint database, and adopted a Markov-chain prediction model to assist positioning [17].

Proximity Beacon is a proximity device designed to be detected by BLE-compatible devices such as Android and IOS smartphones when they are in range of the device. LTE Beacon is a device designed for tracking assets, vehicles, and people both indoors and outdoors; it has the ability to detect nearby assets via Bluetooth, and can be programmed via Javascript [18]. One of the most popular devices on the market is Apple’s AirTag, with a value proposition to prevent personal items from getting lost; however, being part of the Apple ecosystem it only has full compatibility with IOS.

Experimental developments were presented in [19] for pebBLE and Estimote BLE devices, both battery-powered and with a transmit power of 4 dBm, and were subjected to attenuation comparisons for WiFi and BLE. Each signal was measured for one minute at reference distances from 1 m to 13 m with 2 m intervals [19]. Although WiFi has superior overall received signal strength, the attenuation coefficient for BLE is smaller, making it suitable for indoor positioning applications

Phutcharoen et al. concluded in [20] that although BLE technology is widely used for positioning in indoor environments, there is a high error due to the multipath fading that frequently occurs in these scenarios. Their paper presents the study of indoor positioning accuracy in a 91.8 m${}^{2}$ space with three BLE beacons. The fingerprinting technique with root mean square error (RMSE) matching is used to estimate the position of the user equipment. From the results, the average of the measurements can reduce the average distance error to 0.86 m.

The work of Khattak et al. in [21] has tackled the vulnerability problem of fingerprinting frameworks, transforming raw data into a high-dimensional form to better match the existing classification models by utilizing a Bag-of-Features approach. In their work, the cumulative distribution function (CDF) is used in order to better select the proper features based on the positioning distance error [22]. Similarly, Ng et al. proposed a fingerprint database in order to estimate the location of a smartphone user based on a kernel method to define a top-k similarity [23], although in this case the beacons were static and the moving object was in charge of making the estimation. The authors used the CDF to measure the quality of the system.

Table 1 shows a comparison of multiple devices, both commercial and academic, in terms of integrated communications technologies, battery lifetime, use case, SDK, integrated protocols, and integrated sensors.

This work is an extended version of the preliminary results presented in [24], and the contributions can be summarized as follows:

- A complete Hardware–Software architecture for indoor positioning based on fingerprinting is proposed, implemented, and tested.
- An incremental methodology (independent of the deployment scenario) is followed to build a complete dataset for ML-based indoor positioning systems.
- A highly flexible and accurate indoor positioning system is achieved, which can be easily reproduced after being re-trained with the corresponding new dataset.

The rest of this paper is structured as follows: Section 2 presents the proposed general architecture, the development of the scanning stations and TAG devices, and the data classification mechanisms; Section 3 presents the experiments executed in the systems; Section 4 discusses the achieved results; and Section 5 concludes the paper and presents possible future work.

## 2. Materials and Methods

In this section, the proposed architecture of the IoT-based solution for indoor location is be presented along with the corresponding design and implementation process.

### 2.1. General Architecture

**Figure 1:**
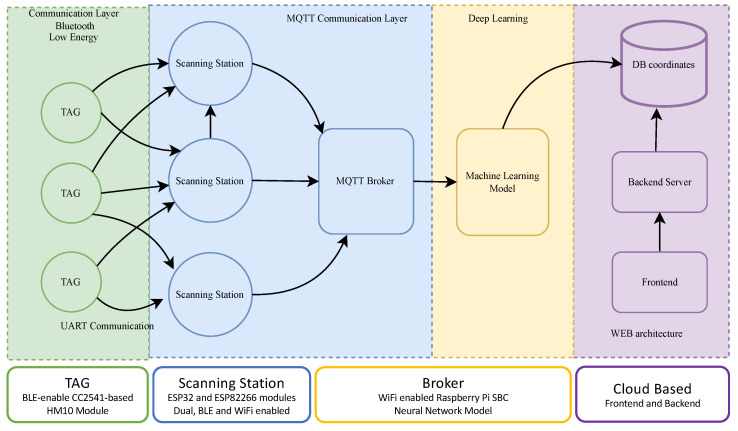
Architecture of the IoT solution for indoor localization based on RSSI fingerprinting (taken from [24]).

The IoT system for indoor asset tracking and identification has as its main objective to locate objects or people that have TAG devices attached or incorporated; its general architecture is presented in Figure 1. The localization of the object is achieved through an ML algorithm with a classification approach based on neural networks, having as the main information the power of the signals received from the TAG in different scanning stations that are distributed in the space under analysis. The information of the X and Y coordinates at the output of the automatic classification algorithm is stored in a database, allowing it to be accessed later from the back-end and front-end to display the data to the end user.

One of the most important contributions of this work is the design, implementation, and deployment of a complete Hardware–Software (HW–SW) solution for indoor tracking of assets. This HW–SW duality can be seen in Figure 1, where the logical structure is presented on top and the hardware components are mapped below. Each is these components is presented in detail in the remainder of this paper.

#### 2.1.1. BLE Layer for TAG Communication

The BLE layer for TAG communication corresponds to the input component of the architecture, involving development of the physical TAG hardware device as well as its firmware. Fundamentally, TAGs are wireless electronic devices with Bluetooth communication capability and long lifetimes. These devices announce their presence periodically (advertising) in a coverage area of up to 15 m. Within the periodic *broadcast* packets, useful information such as the device name, *MAC* address, the manufacturer’s name, and the transmission power can be sent; this information is used later for localization. The TAGs operate in BLE version 4.0 at a frequency of 2.4 GHz and with a configurable power between −23 dBm to 6 dBm. The implemented device has a TI CC254x module (HM10) as its transmission core.

#### 2.1.2. MQTT Communication Layer

As shown in Figure 1, the TAG devices are connected to scanning stations via unidirectional communication, as the stations do not transmit information to the TAGs; however, multiple stations can (and ideally should) receive information from the same TAG. This MQTT communication layer has two fundamental components: the stations and the server.

**Figure 2:**
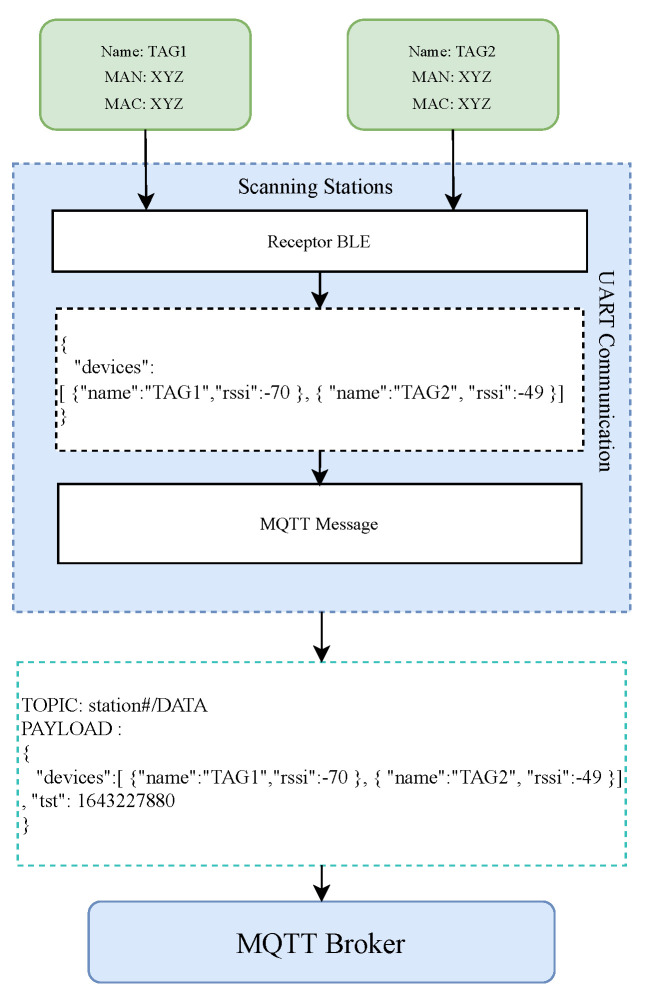
Communication architecture of the scanning stations (taken from [24]).

The scanning stations have the ability to communicate via BLE and WiFi. The BLE functionality of the stations receives the different advertising packets from the available TAGs and selects the relevant information for the application. The WiFi link utilizes the MQTT protocol to communicate with the server, allowing multiple stations to be connected to the same MQTT server and to publish the information received from the TAGs. The data that are published from the TAGs to the server correspond to raw data, with no pre-processing applied in any of these two layers. The subsequent stages are the ones in charge of the position estimation. Figure 2 shows the communication architecture of the stations, from the messages received by the TAGs, the M2M communication between the BLE and WiFi modules, and the conditioning of the frame sent to the server.

#### 2.1.3. Deep Learning Layer

**Figure 3:**
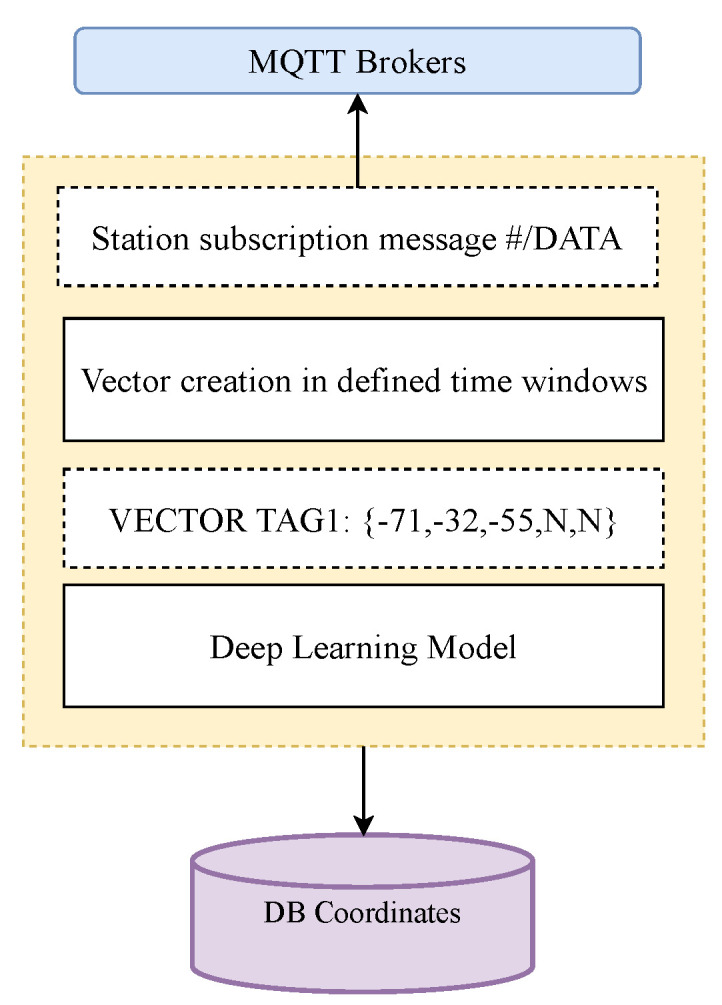
Block diagram representation of the deep learning layer (taken from [24]).

This layer is responsible for taking the RSSI information collected from the TAGs by the multiple stations, passing it to the neural network that has been previously trained in the same environment and under similar conditions, and generating the final result, which is a vector containing the coordinates (label) of the TAG with the corresponding time to be stored later in the database. The operation of the learning layer is depicted in Figure 3, and can be summarizes as follows:

- Connect as a client to the MQTT server and subscribe to topics related to station data.
- Create a vector associated with the data received by the stations in a predefined time window. For each TAG, a packet is generated that includes the corresponding RSSI values received by each scanning station. An “N” character is included in the position of any scanning station that did not receive an RSSI value for that TAG, for instance, VECTOR_TAG_# = [-71,-32,-55,N,N].
- Input the vector to the machine learning system.
- Save the output of the machine learning system to the database.

#### 2.1.4. Web Architecture

The fundamental idea of the web architecture layer is that the end user has the ability to access the location information of the available TAG devices. Therefore, the information coming from the DL module, which corresponds to the TAG coordinates and the time, is stored in the database. The backend oversees the direct communication with this database and the interaction with the user, and the different requests are managed in the frontend by means of HTTP calls. Therefore, in order to obtain different interactions in the future, such as voice assistants, chatbots, SMS, or APIs, different calls to the backend server can be made to find out the information of a specific TAG.

### 2.2. Scanning Station and TAG Devices

The details of the IoT system solution for asset location in a controlled space are presented below, including the different techniques used and the design methodology.

#### 2.2.1. Hardware Design

**Figure 4:**
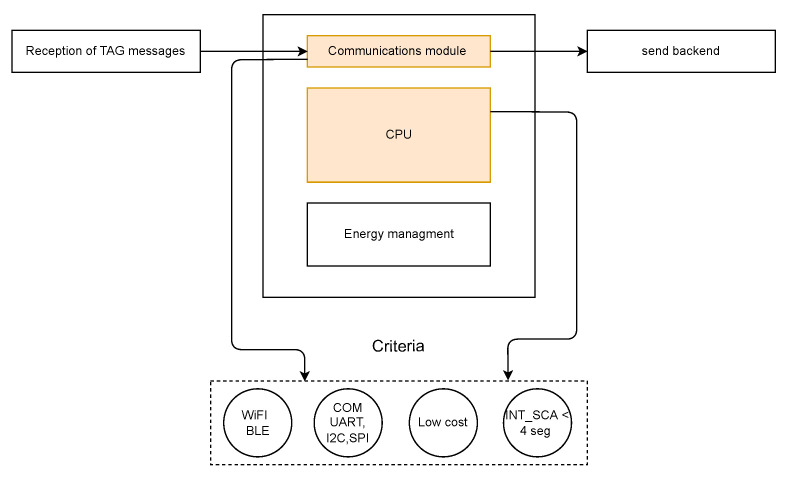
General concept of the scanning station.

The scanning stations play a fundamental role within the system, being in charge of receiving the BLE broadcast packets from TAGs in their coverage area and then sending this information via WiFi to a central server where the position estimation is performed. The stations can perform the scans in two modes, active and passive, the main difference being that in the passive mode no scan request is sent, and therefore certain device information such as the name, device model, manufacturer, and configuration information cannot be obtained. Figure 4 shows the functional block diagram of the scanning station.

In order to have a flexible system, it is desirable that the embedded device has different serial communication protocols available, such as I2C, SPI, and UART, for interfacing with sensors that may be required by the station. For this application, the station should perform scans in the shortest possible time window according to the advertising and system constraints of the TAGs, while understanding that frequent scans directly affect TAG power consumption. Additionally, in order to have a scalable and massive IoT system, the scanning stations and TAGs should be as inexpensive as possible. Table 2 presents and compares commercially available Single-Board Computers (SBCs) that could fulfill these requirements.

According to Table 2, there are different low-cost SBC alternatives that can meet the needs of the system, including Banana Pi, Nano Pi, Neo Air, and Raspberry Pi, all of which integrate both WiFi and BLE, permitting both TAG scanning and information centralization to the server on the same board. During development, tests were performed on a Raspberry Pi Zero W by scanning TAGs and sending data packets to the server, achieving a minimum response time of over 20 s (scanning time window + sending information to the server). With a target time of around 5 s, the BLE scan process was moved to an ESP32 board in order to run it concurrently to the server communication process. The ESP32 board and the Raspberry Pi Zero W were integrated via UART, and a total response time of 4 s was achieved [25].

**Figure 5:**
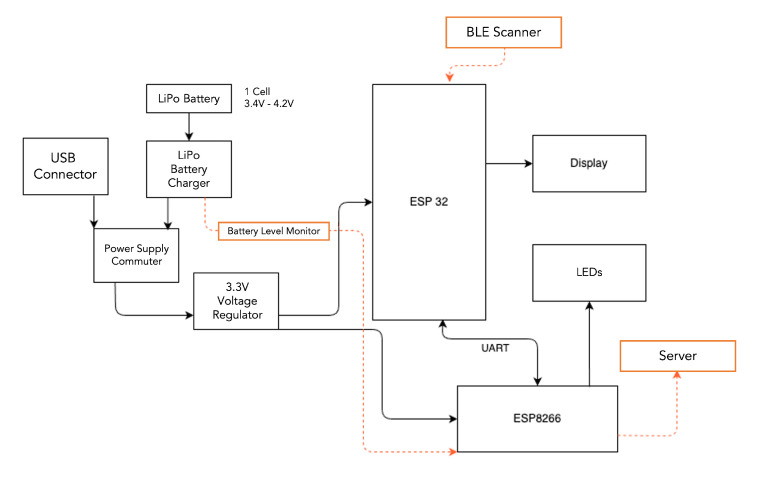
Block diagram of the final design of the scanning station (based on [24]).

Considering that in this scenario the Raspberry Pi Zero W is exclusively in charge of receiving the information through UART and sending it to a MQTT server, we decided to replace it with an ESP8266, taking a lower cost option while retaining the option of having simultaneous BLE scan and WiFi communication. Figure 5 presents the basic hardware components diagram of the final scanning station.

The scanning stations were powered through a 5 V DC supply; in the event they ran out of power, a LiPo battery (TR 18650, 6000 mAh–3.7 V) was used as a backup, allowing autonomous operation for approximately 6 h, for which a *Power Supply Commuter* block was needed. The voltage at the output of the commuter needed to be higher than 3.3 V plus the *dropout* of the regulator; additionally, both components (the commuter and the regulator) needed to be able withstand the power supply current of the circuit. The 3.3 V *Voltage Regulator* block powered the ESP32 and ESP8266 integrated circuits. The communication between the ESP32 and the ESP8266 was via UART, with the *Display* (OLED DISPLAY 128×32) and *LEDs* (RGB) modules providing the mechanism for user feedback and showing the WiFi connection status of the station.

**Figure 6:**
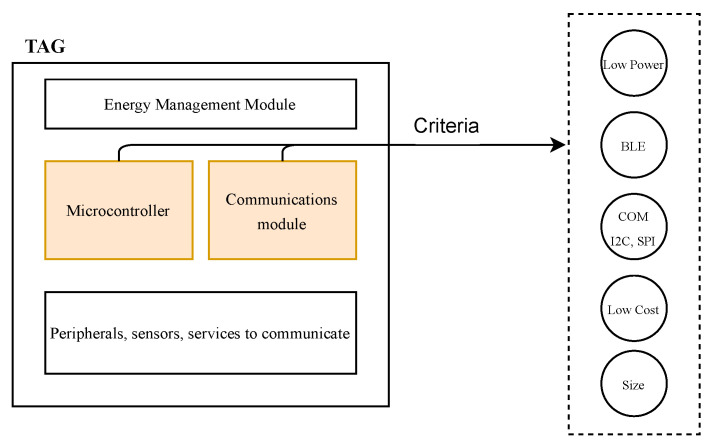
General concept of the TAG device (taken from [24]).

To develop the TAG device, a processing unit and communication stage are required, which are normally integrated in a single module. Figure 6 shows the different elements required for the TAG device, namely, low power consumption, BLE (Bluetooth Low Energy) communication, serial communication, low cost, and small size.

After a review of the BLE principle of operation, the selected module must support the BLE beacon mode (TAG mode) in order to send broadcast messages over the 2.4 GHz channel at different transmission rates (broadcast intervals). The broadcast message is known as the *Advertising Packet*, and the connection with the beacon occurs following the master–slave principle [26]. This mode of operation and the device must allow the transmission of other relevant information, such as the manufacturer’s name and information and the device name, which correspond to the ID when receiving information from the broadcast messages [27].

Solutions with the HM-10 or JDY-23 modules were considered, which have the possibility of being powered with a cell-type battery, and depending on the configured mode of operation (through AT commands) can have lifetimes longer than six months. For the final version of the TAG device, the HM-10 module integrating a TI CC2541 IC was utilized [28].

#### 2.2.2. Firmware

**Figure 7:**
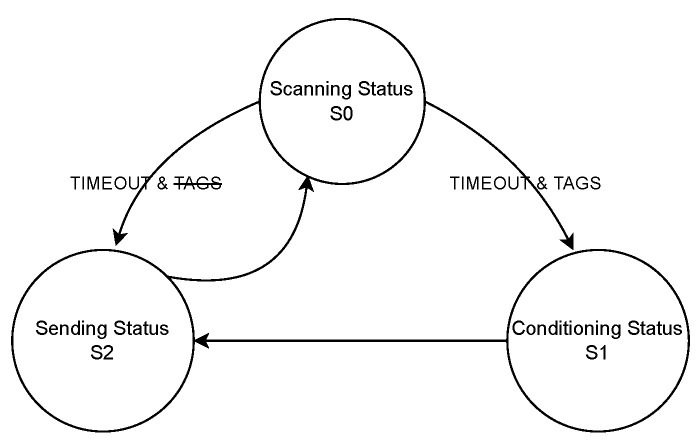
Scanning station FSM diagram for the BLE scanning phase (performed by the ESP32).

**Figure 8:**
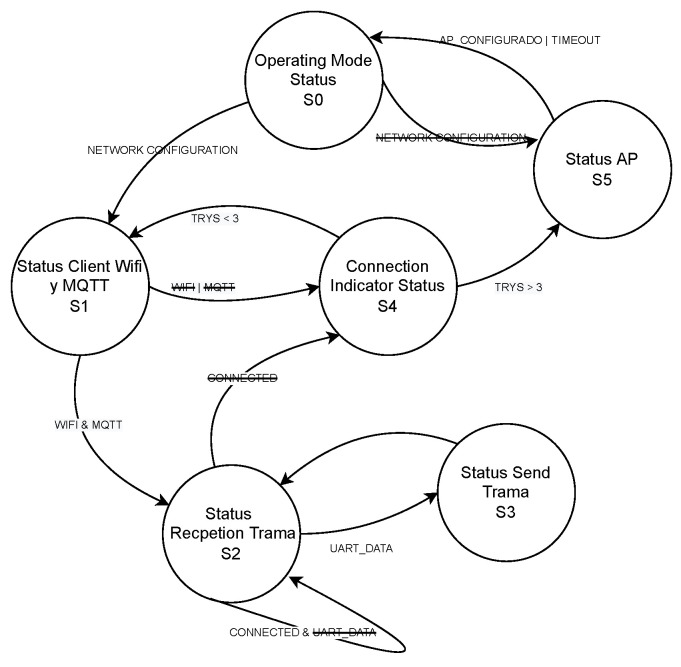
Scanning station FSM diagram for the consolidation of information to the MQTT server (performed by the ESP8266). Taken from [24].

The main goal of this section is to present the development of the firmware architecture (scanning station and TAG) and to document the processes, codes, and information. As previously presented, the scanning stations are composed of two integrated circuits, an ESP32 and an ESP8266, with a common UART interface between them, while the TAG devices integrate an HM-10 module. The scanning station’s firmware was designed following a finite state machine (FSM) approach. Figure 7 shows the FSM diagram for the station’s BLE scanning phase running on the ESP32 module, including the different states and transitions. Figure 8 shows the FSM diagram of the ESP8266, the main function of which is to send the consolidated information from the BLE scanning phase (received via UART from the ESP32) to an MQTT server.

The TAG device is configured in iBeacon mode, performing the *advertising* process in intervals of around 2 s. This particular value was selected as the scanning process runs every 4 s (scan window) and it must be guaranteed that there is at least one scan in each scan window. Of course, as the TAG lifetime depends directly on the broadcast interval and transmission power, it is not recommended to reduce the interval below 2 s.

In terms of programming languages and tools, the TAG devices (HM10 modules) are programmed directly via AT commands (UART interface), configuring them in the corresponding broadcast configuration. The scanning stations (both the ESP32 and the ESP8266 modules) are programmed in C language, integrating different libraries and utilizing the Arduino IDE.

Finally, the neural network model was deployed in the edge device, a Raspberry Pi Zero W running Embedded Linux, with Python 3 as the main programming language and utilizing the Linux Python SDK provided by Edge Impulse to run the machine learning models and collect sensor data (https://github.com/edgeimpulse/linux-sdk-python, accessed on 5 January 2023). The structure of the application was split simply into three parts: MQTT scanning (connection with the scanning stations), vector generation (formatting data for the classifier), and main location classification (based on the Edge Impulse libraries).

### 2.3. Data Classification

In order to identify the position of the different TAGs in a controlled space, a DL classification approach was selected supported by Edge Impulse [29], an ML end-to-end web-based platform. As with any other supervised ML approach, the system must be trained for a specific environment or space of operation, and its operation is restricted to the same location. The general steps for this stage are as follows:

1. Establish and define the architectural plan of the controlled place where the dataset will be acquired and the system performance evaluated.
2. Strategically locate the stations, trying to cover the entire physical space where the tests are to be performed.
3. Establish the input vectors to the automatic learning system to perform both the training data collection and the operation tests.
4. Locate the TAG devices in the established places to collect the training data. Every single location should be associated with a unique label.
5. Train the model for the different input vectors and labels of the system.
6. Perform the output tests of the model, comparing them with the physical space in which they are located.

The collection of the corresponding dataset is one of the most demanding stages of the project, and as such the determination of a correct, independent, unbiased, and representative sample size is paramount [30]. Hence, a reduced experiment was initially proposed with only three locations in order to evaluate the viability of the approach; later, a complete system was built.

#### 2.3.1. Controlled Space under Analysis

**Figure 9:**
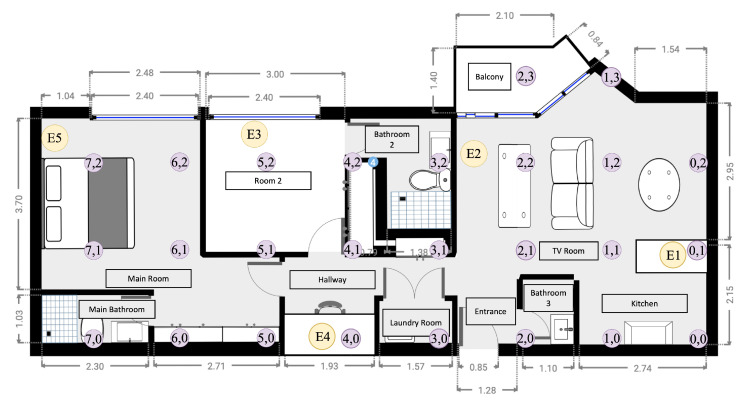
Floor plan of the space under analysis, showing the scanning stations (yellow circles) and predefined locations for the TAGs (labels). Adapted from [24].

The place defined for the system tests corresponded to a two-bedroom apartment with an area of 80.46 m${}^{2}$, a roof height of 2.57 m, and a perimeter of 47.8 m. Figure 9 shows the floor plan of the place in which the different tests were performed; the yellow circles represent the scanning stations and the purple circles are the predefined locations/labels. It should be noted that the arrangement of the stations did not vary either in the tests or during the execution of the system.

Table 3 lists the dimensions and the different characteristics of each space, which depending on the space are relevant because the density of the different materials has a direct effect on the performance of the system.

#### 2.3.2. Scanning Station Location and RSSI Values

**Figure 10:**
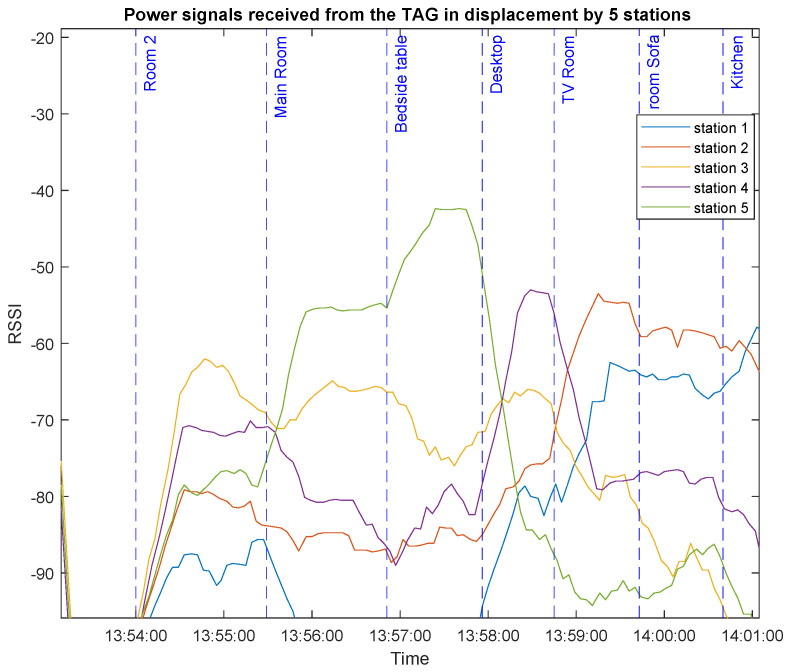
Received RSSI values for the corresponding broadcast messages sent by the TAG at different locations (taken from [24]).

In order to understand and characterize the behavior of the system, a coverage test was performed in the entire controlled space, acquiring the information from a single TAG in the different spaces of the apartment. Figure 10 shows the RSSI values of the broadcast packets received from the TAG for different locations at different times.

As expected, the received power is higher for the scanning stations that are closer to the TAG. However, while the TAG moves from one location to the other, the power intensities vary considerably, making it almost impossible to estimate the location of the TAG. Hence, for these scenarios an intelligent system is required to implement the fingerprinting technique, as explained earlier.

#### 2.3.3. DL Model Input Information and Parameters

**Figure 11:**
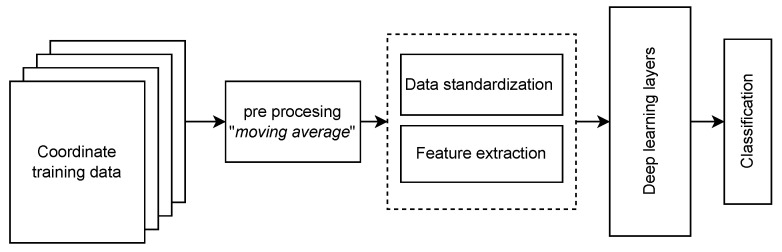
Block diagram of the ML approach followed in this project (taken from [24]).

Figure 11 presents the main elements for the designed and implemented location estimation technique considering the coordinate training data, pre-processing, data standardization, feature extraction, model operation, and classification stages.

- Coordinate training data: corresponding to the files generated for each of the coordinates, where the RSSI values are collected for all stations in a defined time window and formatted as presented in Table 4.
- Pre-processing: corresponding to the moving average filtering technique performed for each of the data groups in order to smooth the signals and deliver them in an cleaner way to improve the model accuracy.
- Data standardization and feature extraction: These blocks already correspond to those implemented in Edge Impulse, in which normalization of the information is performed by scaling them ([−100:0]). In addition, feature extraction is performed by connecting the corresponding processing blocks in the web platform (Edge Impulse).
- Deep learning layers: corresponding to the operation of the previously trained neural network. In a typical neural network, each layer delivers information about the different features of the signal to the following layer, establishing a weight and a bias to deliver the final classification.
- Classification: the output layer of the neural network provides a score for each class, allowing the selection of the most probable label corresponding to the coordinate of the TAG location.

**Table 4:** Example of a sample of the RSSI values of the broadcast packets received by all stations for a defined period of time for location (6,2). Data from [24].

Received RSSI	Timestamp
−61, −69, −100, −93, −100	14/02/2022 14:49:16
−69,−70,−100,−88,−100	14/02/2022 14:49:20
−58,−75,−100,−86,−100	14/02/2022 14:49:28
..., ..., ..., ..., ...	...
−60,−67,−100,−89,−100	14/02/2022 14:49:49
−60,−71,−100,−90,−100	14/02/2022 14:49:53

In order to establish the input data structure for the DL model, different tests were performed with the objective of finding the best parameters for the collection process, specifically, the number of values for the moving average ($N_{X}$) and the time window ($t_{w}$). The value of $t_{w}$ depends on the reception of the messages at the server side, which takes approximately 4 s to consolidate the data and assemble each of the information packets. Therefore, a $t_{w}$ of 40 s would imply taking ten samples from each station for the same instant of time.

Using the Edge Impulse platform, the neural network was trained by altering the input data with different $t_{w}$ and $N_{X}$. Subsequently, an evaluation of the model was performed for only three classes (three locations/labels) and the best combination of pre-processing values were maintained to train the model for all 26 locations. Table 5 shows the accuracy and loss percentage of the DL model for a learning process with 30 learning cycles, a learning rate of 0.0005, and an 80–20% training–validation ratio. Taking into account these results, Table 6 presents the confusion matrix for the case of higher precision ($t_{w}=10$, $N_{X}=2$) for only three locations in the controlled space ((0,0), (1,3), and (2,2)).

#### 2.3.4. Final Deep Neural Network Structure

As presented above, the system was initially trained with labeled data collected on only three locations. However, in order to fully test the system the complete dataset for all 26 locations was necessary to train the neural network model again on Edge Impulse. For each of the 26 selected locations, a one-hour data collection process was performed to create the full labeled dataset. During this time, the TAG was manipulated, rotated, shook, obscured by a hand, etc., to reproduce typical operation conditions of an asset being tracked. These collection conditions of course generate noise in the RSSI signals; this is paramount for the neural network to properly generate a model capable of generalizing. The collected data were later uploaded in JSON format to Edge Impulse.

Different input processing blocks were evaluated (Flatten, Spectral Analysis, and Raw Data); based on the performance achieved with only three locations, Flatten was chosen as the input processing block. Edge Impulse utilizes the Keras library to train and deploy the neural network, offering a suggested structure for the hidden layers.

**Figure 12:**
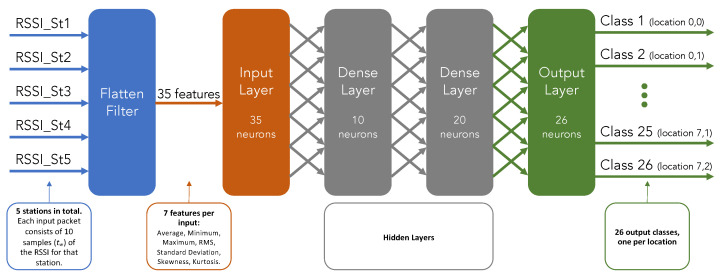
Final structure of the implemented and deployed neural network.

Figure 12 presents the final structure of the neural network that was trained and later deployed. Each input from one station is a time series consisting of the last ten samples of the received RSSI values of the tracked object. With these input signals, for each time series the Flatten filter generates seven features: average, minimum, maximum, root mean square (RMS), standard deviation, skewness, and kurtosis. Hence, considering that five scanning stations have been deployed in the area under analysis (see Figure 9), there are 35 features for the input layer (35 neurons) of the deep neural network. To capture the particular characteristics of each class, the Edge Impulse platform suggests a structure with two hidden dense layers (10 and 20 neurons). Finally, as there are 26 different locations, the output layer has 26 neurons to classify the output into 26 different classes.

A very useful tool available in Edge Impulse is the graphical representation of the generated features before the DL block, which is presented later in Section 3.

#### 2.3.5. User Platform

The user platform corresponds to the back-end and front-end of the system, which is the application layer of final interaction with the user. Different elements of Google Cloud Platform (GCP) Firebase were used for the development of this layer. For this project, the *Firebase Authentication* module was used to identify the user’s information, and the *Firebase Real-Time Database* stores and synchronizes data with a non-relational database hosted in the cloud.

**Figure 13:**
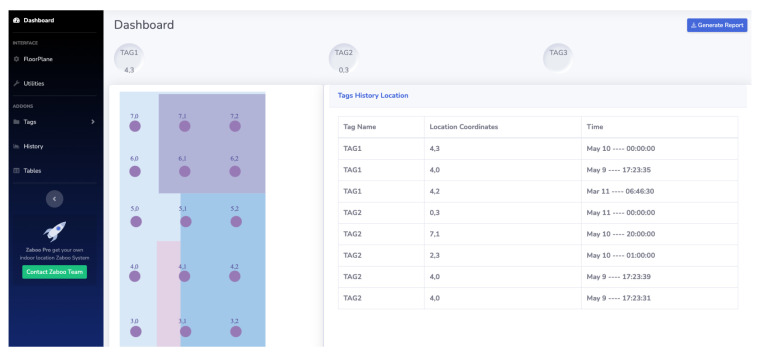
Frontend interface used to present the current location and previous locations of the TAGs.

For this application, HTML5, CSS and Javascript are used to generate the requests to the database and display the graphical user interface in the frontend. The user’s information is stored in documents, and through this a relationship is presented with the stations and TAGs associated with them. The local MQTT client saves the data of the coordinates at the output of the classifications model, information which can be consulted by the user directly in the database after it is authenticated. This platform allows the user to visually identify the location of the TAGs (assets) in the area under analysis. The interface allows the user to see the history of the TAG locations, as shown in Figure 13.

## 3. Results

This section presents the experimental configuration and tests conducted for the different components of the system, as well as for its hardware, firmware, and data classification components.

### 3.1. Hardware and Firmware

**Figure 14:**
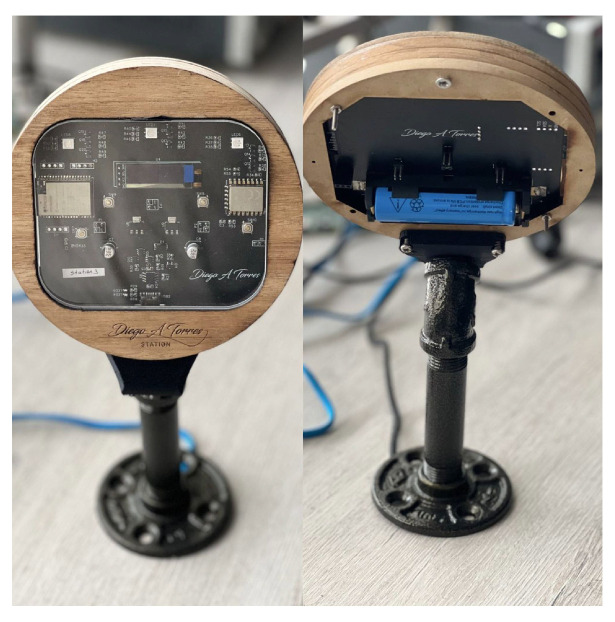
Final implementation of a scanning station (front and back). Taken from [24].

Figure 14 shows the implemented hardware for the scanning stations. For the conducted tests, five scanning stations were assembled and distributed throughout the controlled space as defined in Figure 9. According to the different criteria evaluated in the development of the station, modular tests were performed for the main functionalities (WiFi and BLE communication), as presented in Table 7. Additional tests were performed for the UART on-board communication between the ESP32 and ESP8266 modules.

**Figure 15:**
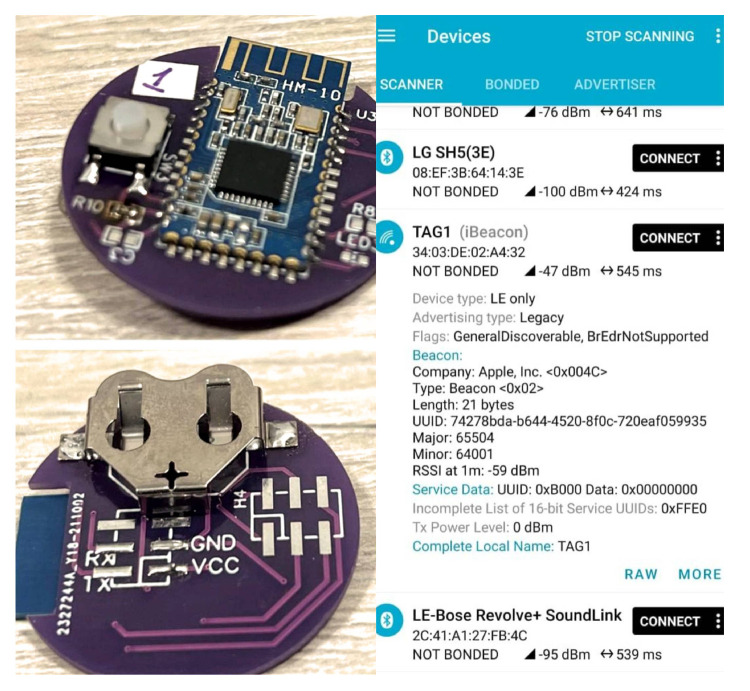
Final implementation of TAG device (front and back) and NRFConnect App (taken from [24]).

Figure 15 shows the final version of the TAG device, including the main components for its operation: the HM10 communications module, the battery holder, and the pins used for the in-board programming. To conduct the TAG performance tests, the mobile application *NRFConnect*, downloadable from Google Play and Apple Store, was used to obtain the information of the BLE devices in the coverage area. For the implemented TAG devices, it was possible to collect the name, configured transmission power, and advertisement interval.

**Figure 16:**
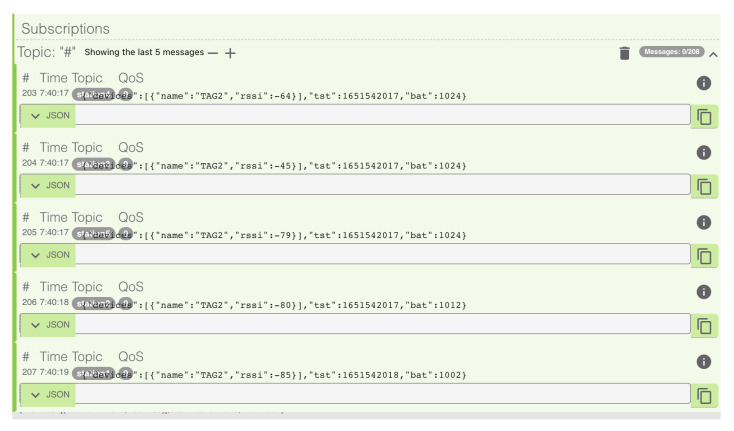
The MQTTLens application, showing the MQTT broker traffic information (taken from [24]).

Finally, a centralization test is required to validate the proper transmission of the TAG information from each of the stations to the MQTT broker. The MQTTLens desktop application was used to observe the traffic messages from the broker, the different messages from the stations, and the corresponding scan times for each of the stations, as shown in Figure 16.

### 3.2. Pre-Processing Mechanisms

**Figure 17:**
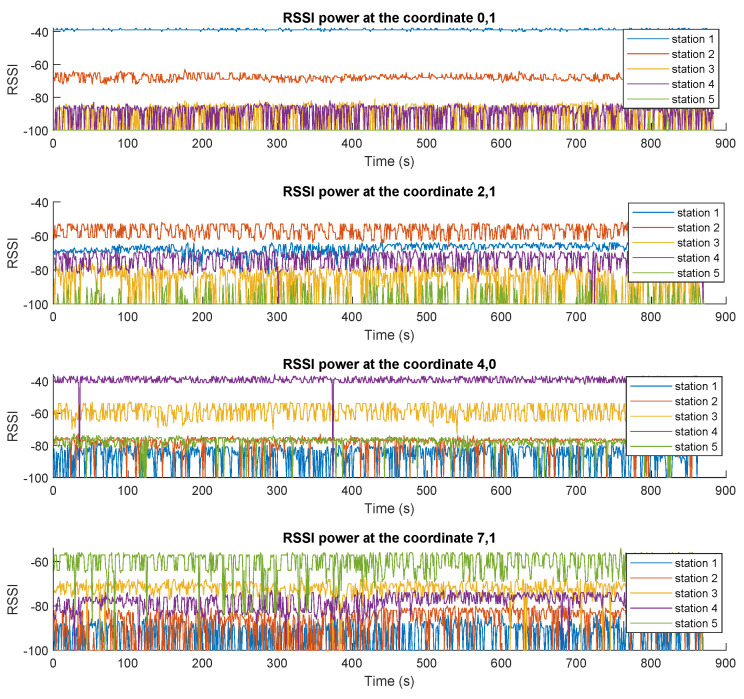
RSSI values received by all five scanning stations for four different locations ((0,1), (2,1), (4,0), and (7,1)).

After the information was consolidated in the MQTT broker and the integration of the system is complete, different tests were performed to observe the dynamics of the received power of the messages coming from the TAGs. Figure 17 presents the received RSSI values for all five stations. The results exhibit several clear trends, with a number of important variations or noise levels that could later affect the performance of the classifier. Naturally, for a fixed position of the TAG, each station receives different RSSI values depending on the distance between the TAG and that station and any obstacles between them.

**Figure 18:**
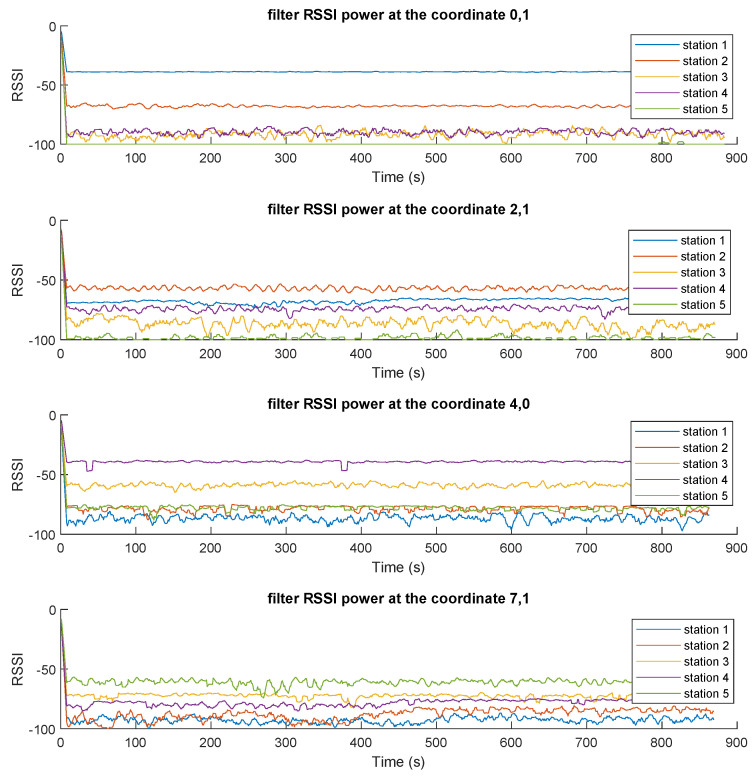
RSSI values after applying the smoothing filter (moving average) for four different locations ((0,1), (2,1), (4,0), and (7,1)).

The noise affecting these signals comes mainly from two sources: (i) on-purpose noise is inserted by manipulating, lifting, obscuring (by a holding hand), rotating, or shaking the TAG in order to reproduce typical operation conditions of an asset being tracked; and (ii) the frequencies at which BLE operates experience natural congestion, as they share the 2.4 GHz band with many devices and appliances, generating collisions, signal superposition, interference, multi-path packets, etc., that affect the received RSSI values. This behavior justifies the need for a smoothing filter, as shown in Figure 18, for which, as presented above, we utilized a moving average of $N_{X}=2$.

After the input signals to the model were established, we proceeded to perform automated data acquisition of the messages broadcast by the TAG, with times of about 90 min for each of the coordinates (labels), while subjecting the TAG to different conditions (rotations, vibration, obstructions, etc.) in order to use data scenarios that do not limit the operation of the system.

### 3.3. DL Input Processing Blocks

A relevant point for position estimation is to define which input processing block should be applied before the neural network in the Edge Impulse platform. Therefore, data grouping, feature extraction, and training tests were performed. The input processing blocks that we considered were:

- Raw Data: input block that considers no pre-processing of the data.
- Spectral Analysis: an input block that is ideal for analyzing repetitive motion, such as data from accelerometers or audio signals, by extracting the frequency and power characteristics over time.
- Flatten: this block flattens an axis into a single value, which is useful for slow-moving averages such as temperature data in combination with other blocks.

Table 8 presents the confusion matrices and F1-scores for the classification system when considering the Raw Data, Spectral Analysis, and Flatten input processing blocks. In these experiments, only data coming from three locations were considered in order to better analyse the separability of the classes when the signal features were generated by the input processing block.

**Figure 19:**
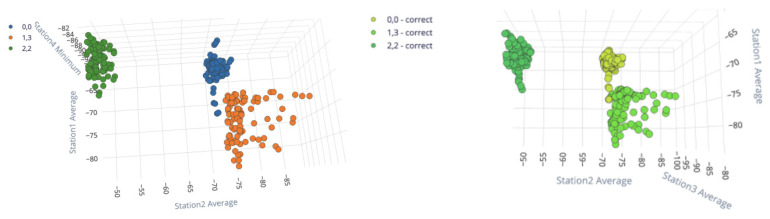
On the left, the features generated by the Flatten input block, with one color per location/label, and on the right the classification results for the same dataset (all in green, all correctly classified). Taken from [24].

Figure 19 shows the features generated after the selected input processing block (Flatten) for a dataset consisting of only three locations ((0,0), (1,3), and (2,2)). As previously explained, this input block is useful for slow-moving averages such as temperature data, or in this case, tracking slow-moving objects. For this block, there is a classification accuracy of 100%, as seen in Table 8, which is the best performance of the considered input processing blocks. In Figure 19, it is important to observe how one class differs from the others, as it is possible to plot hyper-planes after flattening the information on one of the axes; in other words, the classes are separable.

### 3.4. Final Location Classification

**Figure 20:**
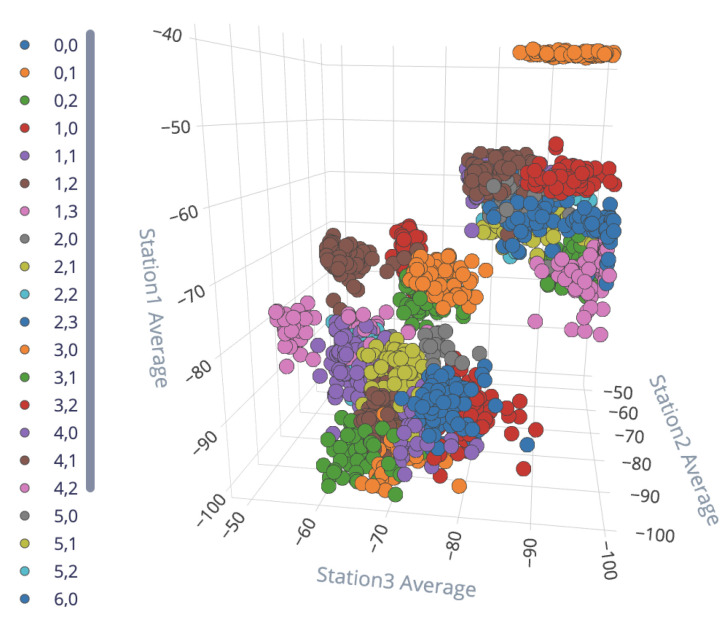
Features generated by the Flatten input block for all 26 locations. One color is assigned for feature datapoints coming from the same location (label).

As mentioned above, Flatten was selected as the input processing block to generate the input features for the neural network to classify. Figure 20 presents the features generated by the Flatten block for datapoints from all 26 locations of the controlled space; the corresponding confusion matrix, precision, recall, and F1-score are presented in Table 9.

Taking into consideration a TinyML approach, the classifier must be executed on the edge without the need to transmit or consolidate the collected information from the TAGs into the cloud. With this in mind, the trained neural network was executed on a Raspberry Pi Zero W implementing the Deep Learning stage shown in Figure 1.

After the model was loaded onto the Raspberry Pi Zero W, about 100 datapoints per location were collected for all coordinates in the area under analysis in order to determine whether the model could correctly estimate the locations of the TAGs in a real deployment. Table 10 presents the prediction of the corresponding coordinates, showing that the model achieved an accuracy of around 88%.

During the test of this deployment, the TAGs were again manipulated (rotated, shook, lifted, obscured, etc.) in order to reproduce similar conditions to real operation of such a system. However, it is worth noting that the TAGs were not moved from one location to another (there are no paths during the tests), and the variations during the manipulation did not significantly modify the location, which was done in order to reduce the complexity of the validation process. Because this was a classification approach, not a regression approach [31], it would be very difficult to define the correct label (location) if the TAG were broadcasting packets while being moved from one location to another.

## 4. Discussion

An accuracy of 88% could be interpreted as having almost 9 correct estimations out of 10 measurements. Even when the TAGs were not moving at all, the system tended to fail in the estimation (1 out of 10 times) because of the highly volatile RF environment. However, considering that the application scenario was that of a tracking system of objects in an indoor environment, it is safe to assume that in a real scenario the objects would not be moving very fast.

With this in mind, a post-processing stage was proposed and implemented which applied a median filter in a window of five datapoints; with this post-processing stage, an increase in the estimation accuracy can be expected. Table 11 presents the accuracy of the classifier with the post-processing stage, achieving 94% on average, though with the disadvantage that it takes more time for the corresponding estimation to be generated.

Within the objectives we set, the system had to be able to send an alert about the presence or absence of targets with a TAG installed. After testing, the solution provides much more information than this, as it is possible to tag many different spaces and know precisely where the object is located.

The use of ML techniques for position estimation solutions provides a great advantage compared to traditional trilateration algorithms, as multiple scenarios and external variables that affect the quality of the estimation can be identified as noise after the corresponding training stage. A relevant point to take into account is that the space in which the system is installed must be previously analyzed, as sometimes locating the stations in the wrong places can affect the estimation process, reducing its final accuracy.

In its current form, the system is trained offline in the Edge Impulse web platform, which in turns generates a static model that can be downloaded (programmed) into the edge device, in this case a Raspberry Pi Zero W. The edge device consolidates the RSSI information from the scanning stations and classifies it accordingly. However, because the neural network model is static, it cannot learn on new labeled data without the need to upload all the data (old and new) and train the model again on Edge Impulse.

For this specific deployment, the above limitation is not a major issue. However, to scale this system it would be preferable to have a base model that can be deployed and a platform that can be retrained based on user feedback, e.g., to correct wrongly classified data, relocate the scanning stations, etc. The development of such a system is part of our future work.

## 5. Conclusions

An IoT System for indoor asset tracking and identification has been designed, implemented, and deployed utilizing a TinyML approach and DL techniques on the edge. We have presented the activities carried out to achieve the different proposed objectives during the development and test stages. In this project, the hardware and firmware for both the scanning stations and the TAG devices were realized. Creative methodologies applied to prior development were used, performing a divergence of ideas and later convergence that led to an optimal development process. System integration was performed as well, involving the scanning of the TAGs by the stations, consolidation of the TAG information in each of the stations, transmission to the MQTT broker, computation at the edge with ML algorithms for position estimation, and finally consolidation to the non-relational database where the data can be consulted by the users.

In this solution, the complete architecture of an IoT solution was developed in which embedded systems concepts were used for the construction of both the scanning stations and the TAGs, which together correspond to the physical layer of the system. In this layer, different M2M and wireless communication protocols such as I2C, UART, and BLE were used. The connectivity layer was designed and implemented for the consolidation of information, in which the MQTT protocol was used to obtain the messages from the broker of each of the stations. The concepts of edge computing and ML were applied to execute a DL model in the embedded system; a storage layer was implemented to ensure remote persistence of the information, and an application was developed that allows visualization of the data.

The cybersecurity in this device contemplates multiple layers that must be taken into account for manufacturing and deployment. Although the architecture was designed to ensure that the information traffic protects the integrity of the data by having only one access point connected to the internet (the broker), the availability and confidentiality of the information could nonetheless be violated. Scenarios of access to the broker and change of data from the payload were considered and basic defense maneuvers were generated, although this is a point to be studied in depth in the future.

The system developed in this project opens up the possibility of continuing the training process after generation of a basic model, making it possible to continue with labeling during the operation of the system through interaction with the user and thereby improving the experience by providing a unique solution tailored to each user. 

## Figures and Tables

**Table 1:** Comparison table of academic and commercial initiatives (taken from [24]).

Device	Sticker Beacon	Proximity TAG	Proximity Beacon	Location Beacon	Location UWB Beacon
Communication	BLE 4.2	BLE 4.2	BLE 5.0	BLE 5.0	BLE 5.0 UWB Beacon.
Battery life	1 year	2 years	3 years	3 years	3 years
Coverage area	7 m	70 m	100 m	150 m	200 m.
Use Case	Asset Tracking	Proximity	Proximity	Indoor Location	Indoor Location
SDK	Estimote SDK	Proximity SDK	Proximity SDK	Indoor SDK	Indoor SDK.
Protocol	iBeacon, Eddystone	iBeacon, Eddystone /URL/UID	iBeacon, Eddystone URL/UID/EID	iBeacon, Eddystone URL/UID/EID	iBeacon, Eddystone URL/UID/EID
Sensors	Accel and Temp	Accel and Temp	Accel, Temp and Light	Accel, Temp and Light	Accel and Temp
**Device**	**LTE Beacon**	**HSKT**	**DWM1001**	**K59**	**TAG STAG220**.
Communication	BLE 5.0	BLE 5.0 UWB	BLE	3.1 GHz–7.0 GHz UWB	BLE 5.0 UWB.
Battery life	2 years	20 h	6 months	5–7 days	5 months.
Coverage area	200 m	100 m	100 m	60 m	10 m.
Use case	Proximity gateway	Health centers	People distancing	Asset tracking	Indoor location.
SDK	WEB IDE	HSKT DK	MDEK1001, Android	SDK k59	–
Protocol	iBeacon, Eddystone	No info	No info	iBeacon	Eddystone URL/UID/EID
Sensors	Accel and Temp	Accel and Temp	Accel	Accel	No info

**Table 2:** Commercially available SBCs (taken from [24]).

Device	Oper. Voltage	Serial Communication	WiFi	BLE	Price
Orange Pi Zero	4.6–5.2	UART, I2C, SPI	YES	NO	$40
Banana Pi P2 Zero	4.8–5.5	UART, I2C, SPI	YES	YES	$50
Nano Pi Neo Air LTS	4.8–5.5	UART, I2C, SPI, GPIO	YES	YES	$18
Rock 64 Media Board	4.8–5.5	RJ45, UART	NO	NO	$180
Pocket Beagle	4.8–5.5	UART, I2C, SPI, GPIO	NO	NO	$60
AML-S905X-CC	4.8–5.5	UART, I2C, SPI, GPIO, PWM	NO	NO	$25
Omega 2	4.8–5.5	2X UART, I2C, SPI, GPIO	YES	NO	$20
VoCore 2	4.8–5.5	3X UART, SPI, I2C, I2S	YES	NO	$17
STM32M P157C	4.8–5.5	UART, I2C, SPI	YES	NO	$22
Raspberry Pi Zero W	4.8–5.5	UART, I2C, SPI, GPIO, PWM	YES	YES	$12
ESP8266 +ESP32	3.3 V	UART, I2C, SPI, GPIO, PWM	YES	YES	$10

**Table 3:** Dimensions and characteristics of the controlled space.

Space	Dimensions	Area	Doors	Windows	Perimeter
Room 2	2.85 m × 3.00 m	8.55 m${}^{2}$	3.32 m${}^{2}$	5.76 m${}^{2}$	10.9 m
Bathroom 2	2.38 m × 2.18 m	3.95 m${}^{2}$	1.41 m${}^{2}$	0 m${}^{2}$	9.09 m
Main room	4.83 m × 5.14 m	16.8 m${}^{2}$	3.13 m${}^{2}$	4.60 m${}^{2}$	18.5 m
Main bathroom	1.03 m × 2.30 m	3.36 m${}^{2}$	1.33 m${}^{2}$	0 m${}^{2}$	6 m
Hallway	1.84 m × 3.61 m	4.93 m${}^{2}$	6.41 m${}^{2}$	0 m${}^{2}$	7.19 m
Kitchen	2.15 m × 2.74 m	4.86 m${}^{2}$	0 m${}^{2}$	0 m${}^{2}$	6.19 m
TV room	4.38 m × 5.39 m	18.4 m${}^{2}$	0 m${}^{2}$	8.07 m${}^{2}$	10.0 m
Bathroom 3	1.40 m × 1.10 m	1.54 m${}^{2}$	1.33 m${}^{2}$	0 m${}^{2}$	4.35 m
Laundry room	0.90 m × 1.57 m	1.41 m${}^{2}$	2.71 m${}^{2}$	0 m${}^{2}$	3.61 m
Balcony	1.62 m × 2.90 m	3.45 m${}^{2}$	0 m${}^{2}$	8.07 m${}^{2}$	4.76 m

**Table 5:** Accuracy and loss in the model for different $t_{w}$ and $N_{X}$ (data from [24]).

$t_{w}$	$N_{X}$	Accuracy	Loss
3	2	53.3%	0.54
	2	40.7%	0.44
5	3	42.9%	0.33
	2	65.6%	0.31
7	3	68.6%	0.35
	4	71.7%	0.43
	2	95.3%	0.17
10	3	91.0%	0.23
	5	90.3%	0.25
	2	76.5%	0.12
15	3	80.6%	0.34
	5	75.2%	0.48

**Table 6:** Model’s confusion matrix and F1-score for the selected locations ((0,0), (1,3), and (2,2)). Data from [24].

%	0,0	1,3	2,2
**0,0**	98.6%	1.4%	0%
**1,3**	11.2%	88.8%	0%
**2,2**	1.4%	0%	98.6%
F1-score	0.93	0.93	0.99

**Table 7:** WiFi and BLE communication tests of the scanning stations (taken from [24]).

Test	Results
WiFi as Client and Access Point	The device generates an offline network with a default password. It is successfully accessed and the network parameters are loaded.
Network Parameters Stored in EEPROM Memory	The device is turned off after loading the WiFi network parameters, and then it is turned on again to validate that the network parameters are properly restored and a connection is available again.
Connection with the MQTT Broker	The device connects to the broker, which is has a fixed IP. The logs register a code 0, which corresponds to a successful connection.
Device BLE Scanning	The scanned devices in the coverage area for the ESP32 are shown in the logs, in a time window of 4 s. Additionally, it is observed in the OLED 128×32 display.

**Table 8:** Confusion matrix for the Raw Data, Spectral Analysis, and Flatten input processing blocks.

Raw Data	0,0	1,3	2,2
0,0	97.0%	2.0%	1.0%
1,3	7.1%	92.9%	0%
2,2	7.5%	0%	92.5%
F1-score	0.91	0.95	0.96
**Spectral Analysis**	**0,0**	**1,3**	**2,2**
0,0	0%	0%	100%
1,3	0%	0%	100%
2,2	0%	0%	100%
F1-score	0.00	0.00	0.52
**Flatten**	**0,0**	**1,3**	**2,2**
0,0	100%	0%	0%
1,3	0%	100%	0%
2,2	0%	0%	100%
F1-score	0.91	0.95	0.96

**Table 9:** Confusion matrix for the classifier using information coming from all 26 locations.

Location	0,0	0,1	0,2	1,0	1,1	1,2	1,3	2,0	2,1
F1-score	0.93	1.00	0.91	0.95	0.87	0.71	0.92	0.84	0.91
Precision	0.87	1.00	0.83	0.97	0.81	0.96	0.86	0.85	0.99
Recall	0.99	1.00	1.00	0.93	0.95	0.56	0.99	0.83	0.85
**Location**	**2,2**	**2,3**	**3,0**	**3,1**	**3,2**	**4,0**	**4,1**	**4,2**	**5,0**
F1-score	0.97	0.98	0.96	0.96	1.00	1.00	0.97	0.90	0.99
Precision	1.00	1.00	0.93	0.98	1.00	1.00	0.98	0.92	1.00
Recall	0.95	0.96	1.00	0.93	1.00	1.00	0.97	0.88	0.98
**Location**	**5,1**	**5,2**	**6,0**	**6,1**	**6,2**	**7,0**	**7,1**	**7,2**	
F1-score	0.86	0.89	0.96	0.76	0.62	0.92	0.95	0.99	
Precision	0.85	0.87	0.95	0.62	1.00	0.93	0.94	1.00	
Recall	0.86	0.91	0.98	0.97	0.45	0.90	0.96	0.99	

**Table 10:** Estimation accuracy of TAG location in real deployment.

Location	0,0	0,1	0,2	1,0	1,1	1,2	1,3	2,0	2,1
Accuracy	86%	87%	87%	85%	88%	90%	89%	87%	88%
**Location**	**2,2**	**2,3**	**3,0**	**3,1**	**3,2**	**4,0**	**4,1**	**4,2**	**5,0**
Accuracy	86%	90%	88%	87%	85%	91%	85%	88%	86%
**Location**	**5,1**	**5,2**	**6,0**	**6,1**	**6,2**	**7,0**	**7,1**	**7,2**	
Accuracy	88%	88%	91%	86%	91%	87%	85%	93%	

**Table 11:** Estimation accuracy of TAG location in real deployment after applying the post-processing stage.

Location	0,0	0,1	0,2	1,0	1,1	1,2	1,3	2,0	2,1
Accuracy	93%	95%	93%	91%	95%	95%	94%	93%	95%
**Location**	**2,2**	**2,3**	**3,0**	**3,1**	**3,2**	**4,0**	**4,1**	**4,2**	**5,0**
Accuracy	92%	97%	93%	90%	92%	94%	89%	94%	89%
**Location**	**5,1**	**5,2**	**6,0**	**6,1**	**6,2**	**7,0**	**7,1**	**7,2**	
Accuracy	95%	93%	99%	94%	97%	95%	80%	97%	

## Data Availability

The data used to support the findings of this study are available from the corresponding author upon request.
